# Clinical implementation of magnetic resonance imaging simulation for radiation oncology planning: 5 year experience

**DOI:** 10.1186/s13014-023-02209-4

**Published:** 2023-02-07

**Authors:** Daniel Moore-Palhares, Ling Ho, Lin Lu, Brige Chugh, Danny Vesprini, Irene Karam, Hany Soliman, Sean Symons, Eric Leung, Andrew Loblaw, Sten Myrehaug, Greg Stanisz, Arjun Sahgal, Gregory J. Czarnota

**Affiliations:** 1 Department of Radiation Oncology, Sunnybrook Health Sciences Centre, 2075 Bayview Avenue, T2, Toronto, ON M4N3M5 Canada; 2 Department of Radiation Oncology, University of Toronto, Toronto, Canada; 3 Physical Sciences, Sunnybrook Research Institute, Toronto, Canada; 4 Department of Medical Imaging, Sunnybrook Health Sciences Centre, Toronto, Canada; 5 Department of Medical Biophysics, University of Toronto, Toronto, Canada

**Keywords:** Magnetic resonance, Clinical workflow, Patient setup, Synthetic CT, MRCAT, Planning magnetic resonance

## Abstract

**Purpose:**

Integrating magnetic resonance (MR) into radiotherapy planning has several advantages. This report details the clinical implementation of an MR simulation (MR-planning) program for external beam radiotherapy (EBRT) in one of North America's largest radiotherapy programs.

**Methods and materials:**

An MR radiotherapy planning program was developed and implemented at Sunnybrook Health Sciences Center in 2016 with two dedicated wide-bore MR platforms (1.5 and 3.0 Tesla). Planning MR was sequentially implemented every 3 months for separate treatment sites, including the central nervous system (CNS), gynecologic (GYN), head and neck (HN), genitourinary (GU), gastrointestinal (GI), breast, and brachial plexus. Essential protocols and processes were detailed in this report, including clinical workflow, optimized MR-image acquisition protocols, MR-adapted patient setup, strategies to overcome risks and challenges, and an MR-planning quality assurance program. This study retrospectively reviewed simulation site data for all MR-planning sessions performed for EBRT over the past 5 years.

**Results:**

From July 2016 to December 2021, 8798 MR-planning sessions were carried out, which corresponds to 25% of all computer tomography (CT) simulations (CT-planning) performed during the same period at our institution. There was a progressive rise from 80 MR-planning sessions in 2016 to 1126 in 2017, 1492 in 2018, 1824 in 2019, 2040 in 2020, and 2236 in 2021. As a result, the relative number of planning MR/CT increased from 3% of all planning sessions in 2016 to 36% in 2021. The most common site of MR-planning was CNS (49%), HN (13%), GYN (12%), GU (12%), and others (8%).

**Conclusion:**

Detailed clinical processes and protocols of our MR-planning program were presented, which have been improved over more than 5 years of robust experience. Strategies to overcome risks and challenges in the implementation process are highlighted. Our work provides details that can be used by institutions interested in implementing an MR-planning program.

**Supplementary Information:**

The online version contains supplementary material available at 10.1186/s13014-023-02209-4.

## Introduction

The evolution of radiotherapy in recent decades has been driven by increases in target dose, target conformity, and delivery accuracy made possible by the introduction of planning computed tomography (CT). That shift from 2D X-ray-based planning, approximately 20 years ago, gave way to subsequent implementation of intensity-modulated radiation therapy (IMRT), image-guided radiation therapy (IGRT), and high-dose stereotactic body radiotherapy (SBRT) [1]. Despite the benefits of CT-based planning, it confers suboptimal imaging for delineating many gross tumour volumes (GTV) due primarily to contrast and resolution limits. Multiple imaging modalities have been used along with CT-based planning to help identify target disease, including ultrasound, PET-scanning, and magnetic resonance (MR) [2].

MR offers several advantages over CT scanning and other image modalities. This includes superior soft-tissue contrast from T1 and T2-weighted imaging, the capacity to assess tumour extent and perfusion with gadolinium-based contrast imaging, and the ability to carry out specialized MR-based imaging such as magnetization-transfer and contrast-enhanced saturation transfer (CEST) imaging for metabolic assessments of tumour extent and response [3]. In general, the method offers a high signal-to-noise ratio and contrast-to-noise ratio with spatial resolutions on the order of 1.5 mm, but it is the differential contrast between tissue types that contributes to its greater utility in imaging over CT-scanning. Due to these advantages, the MR has become the method of choice for improved target delineation in radiation oncology [2, 4].

For the purposes of radiation planning, in most radiation oncology workflows, a diagnostic MR (secondary image) is registered to a planning CT (primary image) using rigid and deformable fusion methods as needed. This process, however, is subject to co-registration errors, where possible sources of registration error include differences in patient positioning, short term physiologically-expected changes in organ shape (such as due to respiratory motion or bladder filling), or target tumour size changes due to the time elapsed between the diagnostic MR and planning CT [5, 6]. Other factors to be considered include that diagnostic MR sequences have been optimized for diagnostic goals, and essential sequences for radiotherapy planning are often absent or have been historically initially available as thick two-dimensional images with low resolution or low geometric integrity [7, 8]. Diagnostic MR sequences, although optimized for disease diagnosis, often require 45–60 min for full image set acquisition, well beyond the 15–20 min available for high-throughput radiation planning for patients set up in immobilization devices. Radiation planning requires the optimization of a subset of pulse sequences—essential to delineate anatomy only rather than full image sets, which may offer otherwise superfluous information.

One of the best alternatives to optimize the use of MR for radiotherapy planning is integrating MR into the planning workflow, known as MR-simulation or MR-planning [9]. Implementing an MR-planning program includes several meticulous processes that ultimately culminate in acquiring optimized MR image protocol for radiation purposes with the patient immobilized as closely as possible to the treatment position [8, 9]. Typical phases of work include having oncologists obtain familiarity with using MR images for tumour identification, optimizing MR sequences to obtain the best possible images while minimizing the time needed for scans, and finally, using MR-planning images for tumour delineation and planning purposes.

In the last 5 years, two dedicated MR platforms (1.5 and 3.0 Tesla) were installed in the Radiation Oncology Department of the Sunnybrook Health Sciences Center, and an MR-planning program was developed and implemented. This report aims to describe in detail the experience in the clinical implementation of MR-planning for external beam radiotherapy (EBRT) and highlight the strategies to overcome encountered risks and challenges so it can be implemented by interested institutions.

## Methods

An MR-planning program was developed and implemented from 2016 to 2021 at the Radiation Oncology Department of the Sunnybrook Health Sciences Center. The clinical implementation is detailed here, including developed workflow, optimized MR image acquisition protocols, MR-adapted patient setup, radiofrequency (RF) coil placement, strategies employed to overcome the encountered risks and challenges, and an MR-planning quality assurance (QA) program. Data on planning site were collected from the treatment planning system (TPS) for all the MR-planning activities for EBRT from July 2016 to December 2021.

For MR-planning, two dedicated MR machines were used. The first was a Philips Ingenia system with a magnetic field of 1.5 T, and the second a Philips Ingenia Elition X system with a magnetic field of 3.0 T. Both scanners were equipped with a 70 cm bore, external laser systems for registration with CT-coordinate systems, and flat and curved tabletops. For MR-safety purposes, the American College of Radiology (ACR) safety zones II-IV were instituted with badge-access required, and a 5-gauss line was demarcated inside scan rooms.

## Results

### Workflow

A workflow was implemented in which the radiation oncologist assigns a care plan (CP) in a patient's chart using MOSAIQ Oncology Information Systems (IMPAC Medical Systems, Sunnyvale, CA). The CP automatically generates a set of pre-defined quality checklists (QCLs) specific for each treatment (i.e., brain radiosurgery) and contains the entire workflow for planning and treatment delivery. Along with the CP, the Radiation Oncologist completes custom fields that include CT/MR-planning scheduling, MR-safety screening, dose/fractionation, and patient setup. A QCL is automatically forwarded to the radiation department that then schedules the CT simulation (CT-planning) and MR-planning, preferably immediately consecutively.

The CT-planning is then carried out according to the physician's order and institutional protocols, ensuring MR-compatible immobilization devices. The MR safety form is re-checked before MR-planning with patients since all patients are required to pass a screening process at least twice. After MR-planning, the radiation therapist proceeds with the CT-MR co-registration, which is later re-checked by the radiation oncologist and/or medical physicist.

### Site-specific setup and imaging protocols

The important sequences for planning MR scans were selected for each anatomical and disease simulation site. The pulse sequence parameters were optimized to meet radiation planning needs, including reducing the voxel size to maximize spatial resolution, reducing slice thickness for optimal resolution, removing or minimizing gaps between slices, and increasing the field-of-view (FOV) for specific protocols (i.e., to include pelvic nodes) [8, 9]. Additionally, one of the essential requirements for planning was to diminish geometric distortion (GD) caused by the non-linearity of gradient magnetic fields and magnetic field homogeneities. The gradient non-linearities are minimal at the magnetic isocenter and gradually increases towards scan limit borders [10]. The GD associated with magnetic field homogeneities depends on factors such as the magnet field strength, patient magnetic susceptibility variation, and image acquisition parameters, specifically the bandwidth [10, 11]. Therefore, image protocols were adjusted to include higher gradient bandwidth in order to provide high geometric fidelity images [10, 11]. All sequences were acquired in the axial plane, and contrast, when needed, was gadolinium-based (Gadavist, Bayer Healthcare Pharmaceuticals, Leverkusen, Germany).

Therapists with dual certification for radiation therapy and MR performed all MR scans. Central nervous system (CNS, namely brain and spine tumours) was the first site to be implemented because it encompassed the highest volume of patients that would benefit from the MR-planning and had the least complex MR imaging process. After sufficient clinical experience with the CNS planning process, gynecology (GYN), head and neck (HN), genitourinary (GU), gastrointestinal (GI), breast, and brachial plexus MR-planning were sequentially implemented every 2–3 months.

The goal of planning MR scans was to replicate the patient's setup on the planning CT. In cases where this is not possible, for instance, due to the limitation of accessories, the patient's positioning and immobilization on MR-planning were made as similar as possible to the CT-planning. Site-specific setups are detailed in Table 1 and summarized further below. Imaging protocols and pulse parameters are described in Table 2. The parameters are detailed for the 1.5 T platform since it was the predominantly utilized machine, and it produces less susceptibility artifacts than imaging at 3.0 T [11].

**Table 1:** Site-specific setups

Disease site	Setup	Image
Setup 1: Brain planning	CT: head board, head support, hybrid 3-point thermoplastic mask, arms on chest, kneefix	
	MR: no immobilization devices are used	
	RF coil: head coil	
	Tabletop: curved	
	Slice thickness: 1 mm	
Setup 2: Head and neck and upper thorax planning	CT and MR: head board, head support, 5-point thermoplastic mask, arms on the chest for all except for by sides for esophagus, kneefix	
	RF coil: anterior, posterior and flex coil for HN and superior esophagus; posterior and flex coil for cervical an upper thoracic spine	
	Tabletop: flat	
	Slice thickness: 1 mm for spine; 1.5–2 mm for head and neck, brachial plexus, and upper esophagus	
	Brachial plexus: patients are shifted toward the contralateral side	
Setup 3: Breast planning	CT and MR: head support, vacuum cushion, arms above the head, align patient’s tattoos to ensure same set up. Vacuum cushion is omitted when it does not fit inside the bore	
	RF coil: posterior and flex coil	
	Tabletop: flat	
	Slice thickness: 1.5–2 mm	
Setup 4: Lower thorax, abdomen, and pelvic bones SBRT planning	CT: whole body vacuum cushion, head support, arms above the head. 4D CT with no abdominal compression for abdominal treatment	
	MR: head support, arms above the head, align patient’s tattoos to ensure same set up	
	RF Coil: posterior coil for spine; posterior and anterior coil for abdominal and pelvic bone SBRT	
	Tabletop: curve	
	Slice thickness: 1 mm for spine; 1.5–2 mm for abdominal SBRT	
Setup 5: Lower thorax and abdomen non-SBRT planning	CT and MR: head support, arms above the head, kneefix, align patient’s tattoos to ensure same set up	
	4D CT-SIM with no abdominal compression for patients with abdominal tumours	
	RF coil: posterior and anterior coil	
	Tabletop: flat	
	Slice thickness: 1.5–2 mm	
Setup 6: Pelvis planning	CT and MR: head support, arms on chest, W foam, align patient’s tattoos to ensure same set up	
	Tabletop: flat	
	RF coil: posterior and anterior coil	
	Slice thickness: 1.5–2 mm	
	Uterine cancer: CT is acquired with full bladder, and MR with both full/empty bladder	
	Vulvar and anal canal: CT-Planning with a vacuum cushion (frog leg); MR-Planning should replicate the frog leg position as much as possible	

*MR* magnetic resonance, *CT* computed tomography, *SBRT* stereotactic body radiotherapy, *RF* radiofrequency

**Table 2:** Image acquisition protocols

Simulation site	Specific protocols	Sequences*	FOV (AP × RL × FH, mm^3^)	Acq Voxel (mm)	Matrix (AP × RL × FH, slices)	TR/TE (ms)	Act WFS (pix)/BW (Hz)	Scan time (min)
Brain	Routine	3D T1 postGad	270 × 270 × 180	1 × 1 × 2	272 × 270 × 180	1.97/6.2	0.390/557	4:53
		3D Flair	250 × 199 × 160	1.15 × 1.1 × 2	216 × 172 × 160	291/4800	0.199/1091.8	5:22
	IAC	Routine + 3D IAC	240 × 240 × 42	0.8 × 0.8 × 1	300 × 299 × 85	2.7/5.4	0.5/434	3:32
	Sella, skull base and meningioma	Routine + 2D T2 FS	220 × 220 × 100	0.8 × 0.8 × 2	276 × 274 × 50	80/4298	0.763/284.8	12:19
		Routine + 3D T1 preGad	270 × 270 × 200	1 × 1 × 2	272 × 270 × 200	1.97/6.2	0.390/557.0	5:39
Spine	Cervical	3D T1	150 × 150 × 100	1 × 1 × 2	152 × 150 × 100	1.96/6.2	0.391/555.6	5:08
		2D T2	180 × 180 × 100	1 × 1 × 2	180 × 173 × 50	90/3404	0.597/363.6	5:37
	Thoracic	3D T1	130 × 130 × 92	1 × 1 × 2	132 × 130 × 92	1.96/6.2	0.390/557.0	4:41
		2D T2	130 × 130 × 92	1 × 1 × 2	132 × 130 × 92	90/3000	0.599/362.8	4:57
	Lumbar	3D T1	180 × 223 × 160	1 × 1 × 4	180 × 223 × 80	1.96/6.1	0.391/555.6	3:08
		3D T2	180 × 223 × 160	1 × 1 × 4	180 × 221 × 80	100/1500	0.344/631.3	3:38
	Sacral	3D T1	240 × 300 × 180	1 × 1 × 4	240 × 300 × 90	1.96/6.1	0.392/554.1	2:48
		3D T2	240 × 299 × 180	1 × 1 × 4	240 × 185 × 90	100/1500	0.438/496	2:03
Head and neck	Routine	3D T1 preGad + postGad	270 × 270 × 255	1 × 1 × 3	272 × 270 × 170	2.1/7.0	0.501/433.5	4:55
		3D T2 FS Head/Upper	270 × 270 × 180	1.2 × 1.2 × 4	224 × 223 × 90	233/2000	0.245/885.7	3:38
		3D T2 FS Neck/lower	270 × 320 × 120	1.4 × 1.6 × 4	192 × 196 × 60	204/2000	0.210/1033.4	5:20
	Nasopharynx	Routine + 3D T2 Nasopharynx	270 × 270 × 180	1 × 1 × 3	272 × 270 × 120	212/2500	0.232/937.8	5:38
Brachial plexus	Routine	3D T1	240 × 300 × 160	1 × 1 × 4	240 × 300 × 80	1.96/6.1	0.392/554.1	3:08
		3D T2 FS	270 × 270 × 140	1.2 × 1.2 × 4	224 × 223 × 70	223/2000	0.245/885.7	4:14
Breast	Routine	3D T1 preGad + postGad	240 × 400 × 240	1 × 1.2 × 4	240 × 334 × 120	1.94/6.1	0.392/554.1	4:48
		3D T1 FS	300 × 400 × 280	1 × 1 × 4	300 × 401 × 140	1.99/3.9	0.328/661.4	10:30
		3D T2	300 × 400 × 240	1.2 × 1.2 × 3	252 × 355 × 160	209/2000	0.293/740.3	4:50
Esophagus	Upper (T5 above)	3D T1 preGad + postGad	200 × 200 × 300	1 × 1 × 4	200 × 199 × 150	2.1/6.9	0.500/434.0	4:18
		3D T2	200 × 200 × 300	1.2 × 1.2 × 4	168 × 164 × 150	220/2000	0.207/1047.9	5:30
	Lower (T6 below)	3D T1 preGad + postGad	280 × 280 × 181	1.5 × 1.5 × 3	188 × 188 × 120	1.72/6.0	0.353/615.6	4:42
		3D T2	300 × 353 × 200	1.2 × 1.8 × 5	252 × 174 × 40	90/2687	0.841/258.3	13:29
Pancreas, liver, kidney	Routine	3D T2	450 × 302 × 210	1.3 × 1.3 × 3.5	340 × 212 × 60	120/5119	0.815/266.4	1:01
		3D T2 FS	300 × 400 × 270	1.4 × 1.6 × 5	216 × 149 × 54	80/878	0.375/578.7	1:35
		3D T1 postGad	450 × 401 × 275	1.5 × 1.7 × 5.5	300 × 229 × 100	1.91/4.0	0.500/434.0	0:15
Prostate	Routine	3D T2	240 × 300 × 160	1 × 1 × 4	240 × 274 × 40	90/1950	1.117/194.5	5:22
	No seed	Routine + 3D T1	240 × 300 × 200	1 × 1 × 4	240 × 300 × 100	1.96/6.1	0.392/554	4:00
	With seed	Routine + SWI Seed	180 × 148 × 75	1 × 1 × 3	180 × 149 × 50	12/51	1.676/129.6	3:31
Gynecology	Routine	3D T2 Full/Empty Bladder	300 × 397 × 270	1.5 × 1.5 × 4	200 × 264 × 135	188/2000	0.219/992.1	4:42
Anal canal, rectum	Routine	3D T1 Pelvis	240 × 240 × 200	1 × 1 × 4	240 × 240 × 100	23/400	0.275/789.1	4:46
		3D T2	300 × 331 × 200	1 × 1 × 4	300 × 331	327/2000	0.328/661.4	4:38

*IAC internal auditory* canal, *SWI* susceptibility weighted imaging, *FS* fat suppression, *GAD* gadolinium, *preGad* pre-gadolinium, *postGad* post-gadolinium, *FOV* field-of-view, *RL* right/left, *AP* anterior/posterior, *FH* foot/head, *acq* acquisition, *SNR* signal-to-noise ratio, *TR* repetition time, *TE* echo time, *WFS* water fat shift, *BW* bandwidth

*All sequences were acquired in axial plane

It was identified that MR-planning was sometimes performed without a prior diagnostic MR and some sequences that could have been useful for diagnostic purposes were eventually absent. To overcome this limitation, additional sequences were added incrementally in the MR-planning protocols (i.e., diffusion) for differential diagnosis purposes. As these sequences are not routinely required for planning, they were not detailed.

### Brain planning

Patients were positioned with no immobilization devices on a curved MR tabletop, using a head coil, similar to the usual diagnostic setup. A hybrid 3-point thermoplastic mask was customized for immobilization for the CT-planning but not used for MR-planning, as the skull allows perfect CT-MR co-registration. This is supported by the literature as there is no difference in image quality or movement artifacts when comparing mask immobilization versus standard diagnostic setup [12]. All the images were kept isotropic in terms of resolution and acquired without angulation.

### Head and neck and upper thorax planning

This section describes the MR-planning of the head and neck, cervical and high thoracic spine, upper esophagus (T5 and above), and brachial plexus. Patients were routinely immobilized for CT-planning with a 5-point thermoplastic mask and arms facing down. This accessory was perfectly compatible with MR-planning, and therefore the setup was identical for both. The posterior, anterior, and flex coils were used for HN, upper esophagus, and brachial plexus, and the posterior and flex coils were used for the cervical and upper thoracic spine.

For spine SBRT, the radiation oncologist defined MR scan limits, which generally included 1–2 vertebrae above and below the target. MR-planning was routinely performed with no contrast; however, it was used in selected cases (i.e., paraspinal disease) at the physician's discretion.

For brachial plexus imaging, patients were shifted toward the contralateral side, whenever possible, to bring the ipsilateral brachial plexus closer to the MR isocentre. Contrast was not routinely required for brachial plexus contouring, but it could be considered for improving tumour delineation at the physician's discretion.

### Breast planning

The MR-planning reproduces the same setup as CT-planning. Patients were positioned supine on a flat tabletop with arms above the head supported by a vacuum cushion (Vac-Lok, CIVCO Medical Solutions). However, the Vac-Lok was omitted in circumstances where it did not fit inside the bore due to patient’s physical habitus. Alignment was based on the skin marks, and flex and posterior coils were used.

### Lower thorax, abdomen, and pelvic bones SBRT planning

This section describes the MR-planning of SBRT treatments of the thoracic, lumbar, and sacral spine, abdomen, and pelvic bones. Patients are routinely immobilized for CT-planning with a whole-body vacuum cushion (Body FIX system, Elekta, Stockholm, Sweden), head support, and arms above the head. This setup, however, was not fully compatible with MR-planning because the Body FIX is too wide and long to fit inside the bore. Therefore, MR-planning was carried out with the patient using no immobilization accessories, with arms above the head. A curved tabletop was used to mimic the shape of the patient's back on the Body FIX and to approximate the patient to the posterior coil, which was especially important for spine SBRT. The posterior coil was used for the spine, while the anterior coil was added for abdominal and pelvic bones SBRT.

As previously stated, contrast was not routinely necessary for spine and pelvic bones but could be used with paraspinal disease at the physician's discretion. Additionally, MR imaging was acquired on free-breath for abdominal tumours to match tomography acquisition.

### Lower thorax and abdomen non-SBRT planning

This topic describes the MR-planning of non-SBRT treatments of the lower esophagus (T6 and below) and abdomen. Patients were generally positioned for CT-planning and MR-planning with head support, arms above the head, flat tabletop, and knee-fix in place. The patient's tattoos were aligned to ensure the same setup. Given that most patients with abdominal tumours underwent planning with four dimensions-CT (4D-CT) and treatment with free-breathing, the MR-planning scan was acquired using free-breathing in order to mimic the averaged scan of the 4D-CT. Anterior and posterior coils were used.

### Pelvis planning

This topic describes the MR-planning of gynecologic, prostate, rectal, and anal cancer. Patients receiving pelvic radiation were positioned similarly for CT-planning and MR-planning. The setup consisted of head support, arms on the chest, and W foam on a flat tabletop. The MR-planning was carried out immediately after CT-planning in order to minimize time elapsed between scans and avoid bladder or rectum filling which would lead to volume differences. Posterior and anterior coils were used.

Particular attention was given to gynecologic and anal tumours. CT-scans of uterine malignancies were acquired with full bladder and MR with both full and empty bladder to account for the dependence of uterus positioning on bladder filling. Patients with vulvar and anal canal neoplasms were positioned on CT-planning with a vacuum cushion for the legs in a frog-leg position, and the MR-planning should replicate the frog-leg position as much as possible. In most cases, this configuration was fully compatible with the MR-planning as the cushion was used on the distal part of the patient's body. The scan typically extended beyond external genitalia inferiorly.

### Challenges in the implementation process

An initial limitation of implementing MR-planning was a shortage of radiation therapists with clinical MR experience, specifically those holding dual MR and radiation therapy certification. Most radiation therapy training programs in Canada still provide only CT-based training; consequently, more than 90% of the department was unfamiliar with MR platforms and did not have any MR background. In order to overcome this challenge, specific recruitment and intensive training were put in place by setting up small learning groups targeted toward each treatment site to address MR knowledge gaps. In the first year of implementation, work was carried out by a limited number of experienced MR technologists with radiation planning background (10 + years) and then supported by newly recruited radiotherapy staff with MR certification but limited MR experience.

Another challenge from a staff perspective was shifting physicians’ culture and mindset from CT-based to MR-based planning. Certain site groups familiar with contouring on MR imaging were more enthusiastic and moved quickly to adopt MR-planning (i.e., CNS and HN). In contrast, other groups used to contouring on CT imaging only were relatively later adopters due to the learning curve of a newly implemented method. Radiation oncologists not habituated to MR images needed specific training in interpreting multiple MR sequences and using them for contouring purposes, which was initially perceived as an increased physician contouring workload. However, this barrier was overcome as they became comfortable delineating on MR imaging, and the benefits of MR-planning became more evident.

We identified a lack of MR safety knowledge among the members of our department. Thus, an MR-safety program was established following the American College of Radiology and Canadian Association of Radiology guidelines. A dual-certified therapist (MR-Radiotherapy) was assigned the departmental role of MR safety officer after acquiring certification from the American Board of Magnetic Resonance Safety. In addition, two Radiation Oncologists acquired formal certification from the American Board of Magnetic Resonance Safety and became MR-Medical Doctor Directors supporting the MR safety program in our department.

We encountered challenges due to the incompatibility of some immobilization accessories, which limited the reproducibility of selected CT-planning setups. For example, the Body Fix is one of the most used accessories for SBRT immobilization, but it is not compatible with MR-planning due to its shape and size. Besides compromising image quality by elevating the patient some centimeters away from the posterior coil and the MR isocenter, the Body Fix is too long and wide to fit inside the bore and under the anterior coil. Smaller vacuum cushions (i.e., Vac-Lok) can be used as an alternative to the Body Fix, but still, the setup is highly dependent on the patient’s physical habitus. In cases where the MR-planning setup was not identical to the CT-planning, we adapted the patient’s positioning in the MR to be as close as possible to the CT, as detailed above and in Table 1.

And lastly, the MR coil selection was challenging when standard uses of coils in a diagnostic imaging department were incompatible with the CT-planning setup. Therefore, in these circumstances, the MR-planning was adapted using non-standard coil configurations to ensure compatibility with the radiotherapy setup and, at the same time, produce diagnostic-quality images. Breast planning is an example that was carried out without a conventional coil placement, as patients need to be prone to use the MR breast coil. Instead, patients were positioned in the supine radiation treatment position with the extremity (flex) coils placed over the breasts. For HN cancer patients, extremity (flex) coils were used along with a torso coil placed on a bridge (holder) above the patient's head, neck, and upper torso to be compatible with the thermoplastic masks. And for prostate cancer, planning was carried out without an endorectal coil.

#### Challenges for patients undergoing MR-planning

Claustrophobia is the most common challenge for patients undergoing MR imaging. Sublingual benzodiazepines are very helpful in facilitating the procedure; however, some patients may have problem completing the planning session despite taking the prescribed medication.

Pain is another prominent issue, as patients can have difficulty maintaining motionless or even completing the MR planning session. This occasionally happens in patients with locally advanced head and neck cancer as they need to stay immobilized in a tight 5-point thermoplastic mask for over 30 min, or with bulky bone metastases. Our policy is to prescribe these patients potent analgesics to provide pain relief. Moreover, MR-planning may be longer than the respective diagnostic MR imaging, which could be challenging for patients in pain. The longer scan times result from thinner slices and higher resolution for MR planning. Therefore, one alternative for patients struggling to complete MR-planning despite analgesics is to streamline MR sequences essential for contouring (omit nonessential sequences) to shorten the image acquisition time.

From the technical aspect, about one-third of our patients have internal medical devices, and often the implant model and MR-safety profile are unknown. When a medical device is present within the patient’s anatomy, we proceed with a cautionary risk–benefit analysis with a multi-disciplinary group of experts (including MR physicists and the radiology department), acknowledging that the main risks include imaging artifacts or implant motion/malfunctioning due to the magnetic field. In situations where MR is essential (i.e., radiosurgery of brain metastases) but could be associated with patient harm, alternatives are considered not to delay immediate treatment. In the case of a not MR-conditional pacemaker, such alternatives can include changing the patient’s device or scanning the patient with constant monitoring in the presence of a cardiology and pacemaker team. Nevertheless, in our experience, most internal medical devices were successfully managed, and the MR-plannings were carried out safely with adequate MR images for contouring purposes.

#### QA program

An MR-planning machine QA program was implemented to ensure safety, operability, and high-clinical performance. Our QA program is aligned with the recommendations of the latest MR-safety guidelines [9] (task group 284 report, American Association of Physicists in Medicine). All test procedures were documented, and results were electronically logged. Relative changes in system performance were assessed by tracking these data and investigating measurements that changed.

The periodic image quality tests (PIQT, Philips) were scanner script-driven and performed weekly. The analysis was automated, requiring only 15 min per session while meeting the National Electrical Manufacturers Association (NEMA) MR standards. ACR large phantom tests were carried out monthly as an independent alternative to PIQT. For monthly 3D geometric uncertainty measurements, evaluations alternated between the Philips Geometric QA phantom and an independent option (Quasar MRD Geometric Analysis System, Modus QA). Relative changes in system performance were observed by tracking these data and investigating measurements that were outside of nominal readings. The detailed QA tests and frequency are described in Additional file 1: Table S1.

#### MR-planning data

**Fig. 1:**
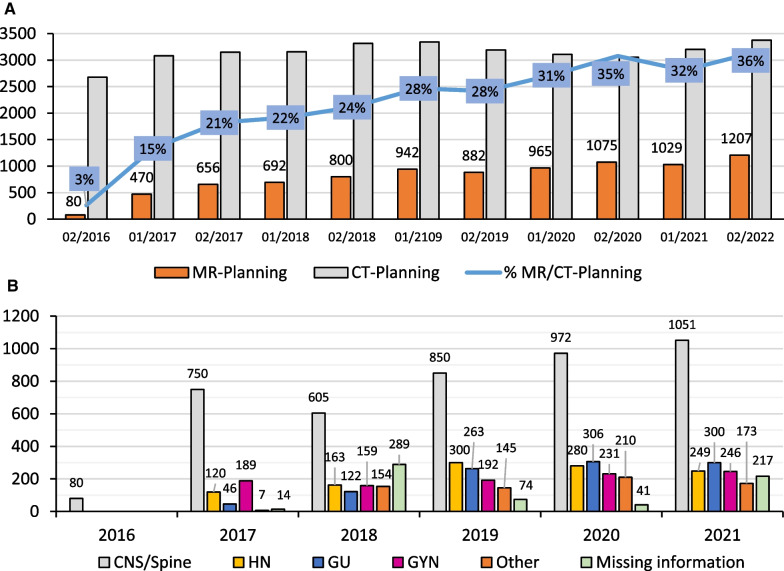
Data of MR-Planning from July 2016 to December 2021. **a** Number of MR-Planning compared to CT-Planning for the same period, per semester **b** number of MR-Planning per year stratified by treatment site

**Fig. 2:**
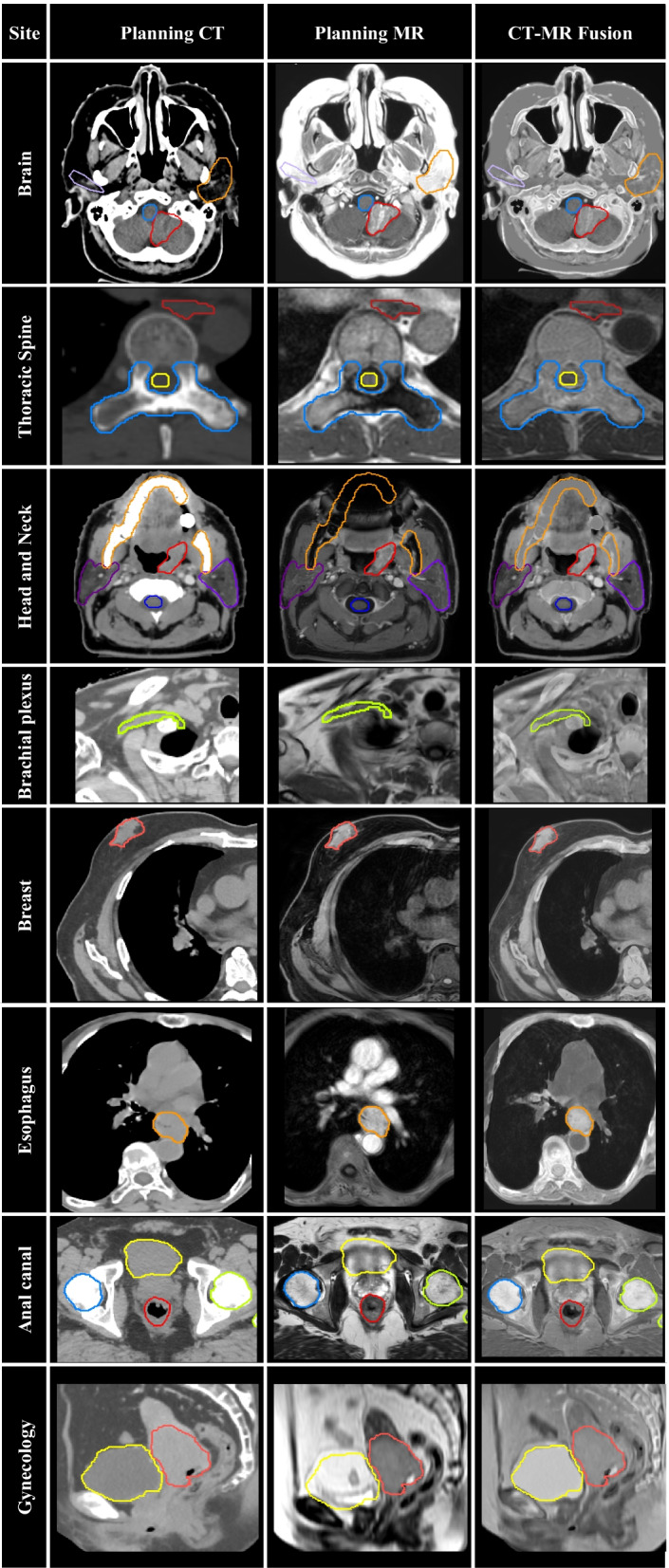
Examples of MR-planning for different treatment sites. (1) Radiosurgery of brain metastasis (red = GTV; blue = brainstem; orange/purple = parotids); (2) Spine SBRT (blue = CTV; red = esophagus); (3) Oropharynx cancer (red = GTV; orange = mandible; purple = parotids); (4) Brachial Plexus (green = brachial plexus); (5) Breast SBRT (pink = GTV); (6) Esophageal cancer (orange = GTV); (7) Anal Cancer (red = GTV; yellow = bladder; blue/green = femurs); (8) Cervical cancer (pink = GTV; yellow = bladder)

From July 2016 to December 2021, 8798 MR-planning sessions were carried out, which corresponds to 25% of all planning CT performed during the same period at our institution. There was a progressive rise from 80 MR-planning sessions in 2016 to 1126 in 2017, 1492 in 2018, 1824 in 2019, 2040 in 2020, and 2236 in 2021. As a result, the relative number of MR/CT-planning sessions increased from 3% of all simulations in 2016 to 36% in 2021. The most common site of MR-planning was CNS (49%), HN (13%), GYN (12%), GU (12%), and others (8%). Figure 1 details the quantity of MR-planning sessions performed since 2016, stratified by treatment-site. Figure 2 provides examples of CT-planning and MR-planning and their co-registration for different treatment sites. Selected contours are presented on the CT and MR to demonstrate image registration accuracy.

## Discussion

We described the clinical implementation of MR-planning for several treatment sites along with relevant technical details. Our study is significant as it details protocols and processes developed in-house to deliver radiation planning scans at diagnostic quality, improved over 5 years of experience, and with almost nominally nine-thousand MR scans. Although there are guidelines and recommendations for the development and implementation of a MR-planning program [8, 9, 13], and image acquisition protocols are available for specific sites [4, 14–22], very few reports have focused on describing clinical implementation experience with comprehensively detailing MR-planning programs [23].

Our report is unique in addressing protocols for planning MR of sites that are not commonly detailed in the literature, such as breast or brachial plexus, and covering almost all tumour locations in the body. Even though planning MR is not routinely used for adjuvant breast radiotherapy, there has been growing interest in MR for partial breast irradiation, breast SBRT, and brachial plexus recurrences [16, 18, 19, 24–26]. For instance, planning MR has been performed in our institution as part of the ongoing phase I/II trial of SBRT for breast cancer patients who are inoperable or refuse breast surgery (ClinicalTrials.gov, NCT03585621). In addition, planning MR is helpful in disease visualization and improving brachial plexus contouring, which has the potential to reduce radiation-related plexopathy of high-dose radiotherapy for recurrent breast cancer, metastatic axillary or supraclavicular nodes, or apical lung tumours [27, 28].

One of the strengths of our methodology was the sequential implementation of a single treatment site every 2–3 months, starting with the most straightforward planning processes and progressively moving towards more complex ones. This allowed the department and staff members to gain expertise and adjust strategies longitudinally. For each scan protocol, the reconstructed MR slice thickness was set up for optimal signal-to-noise ratio and time using standard MR-scanner interpolation to avoid gaps between the slices and to permit compatibility for co-registration. Moreover, the radiology department was consulted as needed to ensure that the pulse parameters being implemented for MR-planning were at the same time optimized for radiation purposes and met diagnostic quality requirements.

There are several benefits of implementing an MR-planning session [2, 9, 29, 30]. Performing CT-planning and MR-planning in identical or similar patient setups is essential to mitigate body and organ shape differences due to positioning. The variability of organ volume and filling for abdominal and pelvic tumours can be minimized as planning CT and MR are also carried out in a sequence with a minimum interval time between scans. The MR-planning guarantees that the necessary sequences are available at high-resolution, high-geometric fidelity, with thin slices with a good signal-to-noise ratio, allowing accurate co-registration and contouring. Furthermore, an otherwise traditional diagnostic non-planning MR acquired outside of the planning process has the disadvantage of delineating a tumour usually scanned weeks before the planning CT. On the other hand, the use of MR-planning shortens the interval from the contouring MR to the treatment initiation, which can minimize geographic misses secondary to tumor growth.

Additionally, incorporating MR in planning workflow goes beyond improving contouring. Recently, MR has been integrated into radiotherapy platforms in place of the cone-beam-CT, namely MR-Guided Linear Accelerators (MR-LINAC), and treatment delivery on such technology has become online MR-guided [2, 31, 32]. Implementing an MR-planning program is the first step in the shift from CT-guided to fully MR-guided radiation therapy, and advantages could include integrating functional MR images and artificial intelligence (AI) to use image texture analysis and develop biomarkers to predict response before and during treatment [33, 34]. It is hypothesized that these types of MR data could be utilized, for example, to adapt radiation dose during treatment depending on tumour response, for example, delivering higher doses to radioresistant regions and lower doses to the radiosensitive segments, an approach known as dose painting [35–37].

**Fig. 3:**
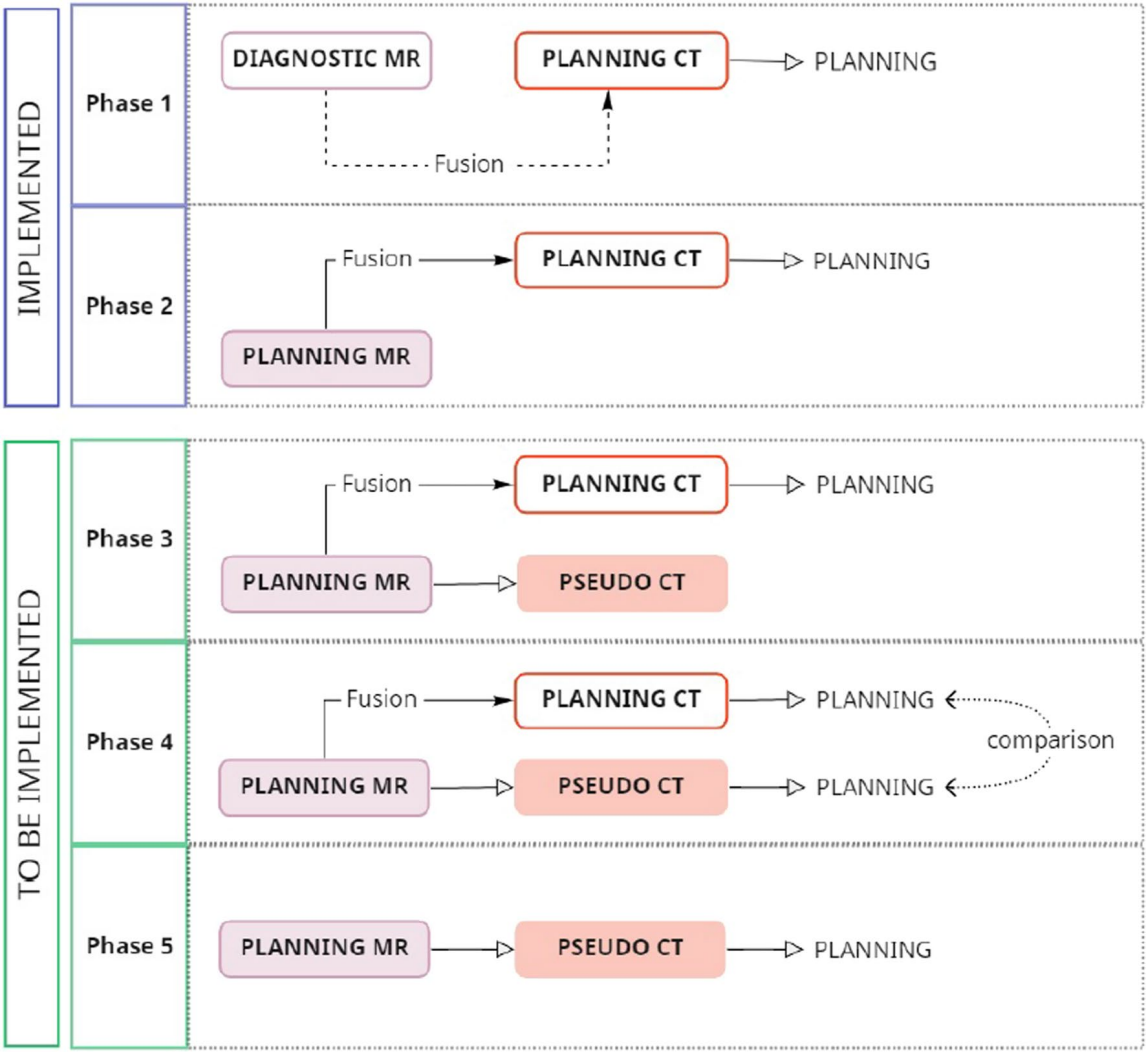
Implementation phases for MR-only workflow. Abbreviations: *MR* magnetic resonance, *CT* computed tomography

**Fig. 4:**
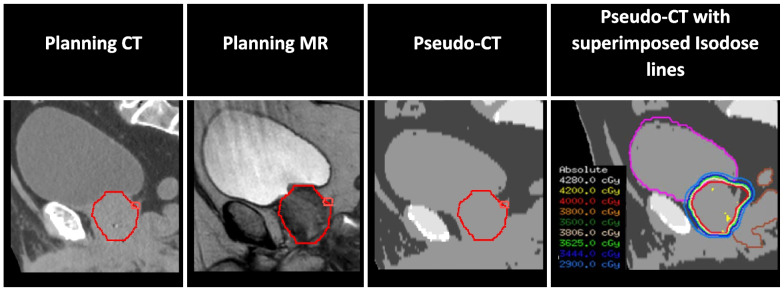
The MR-planning was used to generate a pseudo-CT for treatment planning and dose calculation (red = prostate; pink = seminal vesicles; magenta = bladder; brown = rectum)

As radiotherapy progressively moves towards MR-based treatment, the next step would be to eliminate the planning CT in cases where it is not needed for target definition. The greatest challenge would be using MR alone for dose distribution calculation. The solution relies on algorithms developed to generate a synthetic CT (pseudo-CT) from MR-planning scans for dosimetric calculation purposes, a process known as MR-only workflow [38]. The gain of dispensing the planning CT would include extinguishing registration uncertainty and saving time and resources. As future steps, our department has been studying pseudo-CT for treatment planning and dose calculation, and the goal is to progressively move towards MR-only workflow, as detailed in Fig. 3 and exemplified for prostate radiotherapy treatment in Fig. 4.

In conclusion, we describe processes and protocols developed in-house for the clinical implementation of an MR-planning program for several sites, which has been improved over 5 years of robust experience. Finally, the strategies to overcome the challenges were addressed, and the strengths of our process were discussed. Our work provides details that can be used by institutions interested in implementing an MR-planning program.

## Supplementary Information

**Additional file 1. Table S1**: Quality Assurance Program for MR-Planning. Abbreviations: PIQT, Periodic Image Quality Test; SNR, NEMA, National Electrical Manufacturers Association; ACR, American College of Radiology; MR, magnetic resonance; RF, radiofrequency.

## Data Availability

Research data are not available at this time.

## Abbreviations

MR: Magnetic resonance
MR-planning: MR simulation
EBRT: External beam radiotherapy
CNS: Central nervous system
GYN: Gynecologic
HN: Head and neck
GU: Genitourinary
GI: Gastrointestinal
CT: Computer tomography
CT-planning: Computer tomography simulation
IMRT: Intensity-modulated radiation therapy
IGRT: Image-guided radiation therapy
SBRT: Stereotactic body radiotherapy
GTV: Gross tumour volumes
CEST: Contrast-enhanced saturation transfer
RF: Radiofrequency
QA: Quality assurance
TPS: Treatment planning system
ACR: American College of Radiology
CP: Care plan
CT-planning: CT simulation
FOV: Field-of-view
GD: Geometric distortion
4D-CT: Four dimensions-CT
PIQT: Periodic image quality tests
NEMA: National Electrical Manufacturers Association
ACR: American College of Radiology
MR-LINAC: Magnetic resonance-guided linear accelerator
AI: Artificial intelligence
Pseudo-CT: Synthetic CT
