# Supplementary material for: Clinical implementation of magnetic resonance imaging simulation for radiation oncology planning: 5 year experience
Source: Radiat Oncol. 2023 Feb 7;18:27. doi: 10.1186/s13014-023-02209-4 (PMC9903411; doi:10.1186/s13014-023-02209-4)
Supplement: Supplementary file 1 — Additional file 1. Table S1: Quality Assurance Program for MR-Planning. Abbreviations: PIQT, Periodic Image Quality Test; SNR, NEMA, National Electrical Manufacturers Association; ACR, American College of Radiology; MR, magnetic resonance; RF, radiofrequency. [file 13014_2023_2209_MOESM1_ESM.docx]

Supplementary Table 1:

| Type | Frequency | Test | Description |
| --- | --- | --- | --- |
| CLINICAL | Weekly | Central Frequency Stability | Magnetic field resonance frequency should be stable (automated Philips PIQT test). |
|  |  | Transmitter Gain Stability | Flip angle calibration process should be stable (automated Philips PIQT test). |
|  |  | Signal-to-Noise Ratio | Signal-to-Noise Ratio should be stable (automated Philips PIQT test). NEMA procedure (NEMA MS 1-2008, Method 4) is followed, using head coil and homogenous section of phantom. |
|  |  | Image Uniformity Assessment | Image Uniformity should be stable (automated Philips PIQT test). Using head coil and homogeneous section of phantom, max and min pixel intensity are measured and NEMA Integral Uniformity Measure is calculated. |
|  |  | Geometric Uncertainty 2D | Spatial linearity should be stable (automated Philips PIQT test). Using a hole pattern in the phantom, eight radial distances are measured and maximum absolute differential spatial linearity (%) is calculated. |
|  |  | Spatial Resolution | Spatial resolution should be stable (automated Philips PIQT test). Using the width of line spread function, measured from the profile of an edge in the phantom, horizontal and vertical pixel size are calculated. |
|  |  | Slice Profile and Thickness | Slice profile and thickness should be stable (automated Philips PIQT test). Using a thin ramp in the phantom, three metrics are measured: full-width at half-maximum (FWHM), full width at tenth maximum (FWTM) and Slice Integral. |
|  | Monthly | Percent Signal Ghosting | Ghosting level should be below ACR recommended level (ACR MR phantom). |
|  |  | Low Contrast Object Detectability | Contrast-to-noise ratio should meet the ACR criteria (ACR MR phantom). |
|  |  | Geometric Uncertainty 3D | 3D distortion vector field should remain stable and consistent with commissioning data. Two large field-of-view phantoms are scanned on alternate months, namely, the automated Philips distortion phantom and the Modus MRID phantom, the latter calculates gradient non-linearity and B0 coil homogeneity components. |
|  |  | External Laser Positioning System | The MR-simulation external laser system should accurately (within ±2mm) be able to transfer laser defined isocentre position to the MR isocentre (Philips laser phantom test). |
|  |  | RF Coils: Signal-to-Noise Ratio of Coil Elements | Signal-to-Noise Ratio of each RF coil element should be stable (Philips automated procedures for each clinical coil). |
| SAFETY | Daily | Safety Signage | ‘Magnet On’ sign should be lit. |
|  |  | Cryogen System | Helium level should be stable and compressor chirp should be heard. |
|  |  | Temperature/Humidity | Magnet room and technical room temperature and humidity should be within normal range. |
|  |  | Ferromagnetic Detectors | Hand wand and door-mounted detectors should be functioning. |
|  |  | Room Survey | Console, technical and magnet rooms should be in normal state. |
|  | Monthly | Emergency Electrical Power Off | Emergency electrical power off switch should be functioning. |
| OPERABILITY | Daily | Intercom System | Two-way audio communication should be functioning. |
|  |  | Video Monitor | Monitor should be functioning. |
|  |  | Bore Lights | Bore lights should be functioning. |
|  |  | Bore Fan | Bore fan should be functioning. |
|  |  | Lasers | External lasers should be aligned with reference tape markings on the wall. |
|  |  | Computer Memory | Sufficient disk space should be available to save patient data. |
|  | Monthly | RF Door Copper Finger Check | Magnet room door should have unbroken copper fingers. |
|  |  | RF Coil and Accessory Check | RF coils, anterior coil bridge and MR table including connector sockets should not be damaged. |
|  |  | MR Bore Check | MR bore should not have any loose metallic objects, such as debris/iron fillings. |
|  |  | Clinical Sequences Backup | Latest versions of clinical sequences are backed-up. |
